# Total bilirubin level may be a biomarker of nephropathy in type 2 diabetes mellitus: A meta-analysis of observational studies based on MOOSE compliant

**DOI:** 10.1097/MD.0000000000005765

**Published:** 2017-01-10

**Authors:** Dan Zhang, Bo Zhu, Wei Zhang, Wei Wang, Dan Guo, Ligang Yang, Lu Wang

**Affiliations:** a Department of Endocrinology, Fourth Hospital of China Medical University, Shenyang; b Department of Cancer Prevention and Treatment, Cancer Hospital of China Medical University/ Liaoning Cancer Hospital & Institute Shenyang, People's Republic of China.

**Keywords:** bilirubin, diabetes mellitus, meta-analysis, nephropathy

## Abstract

Recently, the number of the studies on the relationship between the total bilirubin level (TBL) and diabetic nephropathy (DN) is increasing, but their results were not consistent. Therefore, we performed a meta-analysis to analyze the relationship between TBL and the risk of DN.

We searched 5 databases before October 31, 2016, and reviewed the reference list of relevant articles. The fixed or random-effects model was used to pool risk estimates. We conducted the dose–response meta-analysis to evaluate the relationship between TBL and the risk of DN.

Our meta-analysis showed that TBL in the DN group was lower than that in diabetes without the kidney disease (NDN) group (standard mean difference [SMD]: −0.63, 95% CI: −0.80, −0.46). The result of each subgroup also showed that TBL in the DN group was lower than that in the NDN group. The result of meta-regression indicated that duration of diabetes mellitus might be the source of heterogeneity. Our meta-analysis also showed that there was a significant negative relationship between TBL and the risk of DN (OR: 0.86, 95%CI: 0.82, 0.90). The results of subgroup analysis were similar to those of SMD; no sources of heterogeneity were detected by meta-regression. Sensitivity analysis indicated that the results were robust. We observed a linear association between TBL and the risk of DN, and there was a negative dose–response association between TBL and the risk of DN.

In conclusion, bilirubin may be used as a biomarker of DN. It helps early diagnosis and effective therapeutic strategies on DN.

## 1 Introduction

Type 2 diabetes mellitus (T2DM) is a common syndrome with disordered metabolism and endocrine, characterized by insulin resistance and beta-cell dysfunction. In 2012, the prevalence of diabetes was 8.3% all over the world.^[1]^ The Centers for Disease Control and Prevention (CDC) reported that the prevalence of diabetes in the United States increased from 4.9% in 1990 to 11.3% in 2010, increased by 2.31 times.^[2]^ Type 2 diabetes not only caused serious harm to human health, but also brought huge economic burden for individuals and countries. Diabetic nephropathy (DN) is one of the most common and severe microvascular complications of diabetes mellitus. Once diabetic patients develop DN, pathological changes and condition often show progressive trends, cannot be easily reversed. In the United States, almost 40% patients with end-stage renal disease (ESRD) were developed from DN; DN was also the primary cause of ESRD in China.^[3,4]^ The early diagnosis and effective therapeutic strategies on DN have become particularly important, so novel biomarkers with the ability to predict DN progression accurately is needed in clinic practice.

So far, the etiology and pathogenesis of DN is not very clear. Oxidative stress has been associated with the pathogenesis and progression of DN, and it is suggested that antioxidants might inhibit the progression of kidney dysfunction. In animal models, antioxidants have been shown to be effective in treating DN.^[5,6]^ Bilirubin was an important factor of the endogenous antioxidant system in the human body.^[7,8]^ As the bilirubin level decreased, antioxidant capacity fell down. Several studies had shown that there was a negative relationship between the total bilirubin level (TBL) and diabetic microvascular complications.^[9–11]^ Recently, a growing number of studies had focus on the association between TBL and the risk of DN, but inconsistent results had been reported. For example, Toya et al^[12]^ found that no significant increase in patients with DN compared to diabetes without kidney disease (NDN), whereas Hamamoto^[13]^ found that TBL in NDN was higher than that in DN, and confirmed the inverse relationship. Currently, we found no meta-analysis on the relationship between TBL and the risk of DN, so we first systematically reviewed the observational studies and quantitatively assessed the association between TBL and the risk of DN.

## 2 Materials and methods

### 2.1 Search strategy and study selection

In this meta-analysis, we comprehensively searched relevant studies in multiple databases (PubMed, Web of Science, EMBASE, CNKI (China National Knowledge Infrastructure), Wanfang database, and VIP) before October 31, 2016. The search terms were used: “bilirubin” and “diabetes” and (“nephropathy” or “kidney disease”), (The filter: (bilirubin [Text Word] AND diabetes [Text Word]) AND (nephropathy [Text Word] OR kidney disease [Text Word])). The search was restricted to human studies. No restrictions were imposed on language. In order to find more relevant studies, the reference lists of included studies were also searched.

### 2.2 Inclusion and exclusion criteria

DN was defined as the urinary albumin-to-creatinine ratio (ACR) ≥30 mg/g, and contained microalbuminuria and macroalbuminuria. ACR of microalbuminuria was 30–299 mg/g; ACR of macroalbuminuria was equal to or higher than 300 mg/g.

Eligible studies were included by the following criteria: (1) the relationship between TBL and DN should be investigated; (2) patients with diabetic nephropathy were enrolled; (3) the study contained either the effect of bilirubin on the incidence rate of DN in T2DM or TBL in the DN group and the NDN group; (4) the study should report the mean value with standard errors or odds ratio (OR)with 95% confidence intervals (CIs) on the relationship between TBL and DN, or provide necessary data to calculate them. (5) if more than 2 studies came from the same population, the latest or highest-quality study was selected.

The studies were excluded by the following criteria: (1) the study did not report the relationship between TBL and DN; (2) the study was not original research, for example editorial, commentaries and reviews; (3) the study did not involve humans, for example, animal experiments, chemistry, and cell-line studies; (4) the study did not provide sufficient data to calculate the mean value with standard errors or OR with 95% CIs.

Two reviewers (DZ and WZ) independently carried out the process of the study selection and exclusion; the third reviewer (BZ) resolved any disagreements by discussion or consultation.

### 2.3 Data extraction

According to the inclusion and exclusion criteria, the following information was independently extracted from the included studies by 2 reviewers (DZ and BZ): first author's name, published year, the design of study, number of subjects, gender, age, body mass index (BMI), duration of diabetes mellitus and TBL. The units of TBL were different among the studies and used by μmol/L and mg/dL. In order to do the statistics and analysis expediently, we converted mg/dL to μmol/L with multiplying by 17.1. We extracted or calculated the mean value with standard errors or OR with 95% CIs from each included study. We extracted risk estimates with the most adjustment (when available). For dose–response analysis, the following information was also needed: (a) the risk estimates and their corresponding 95% CIs for at least 3 exposure categories; (b) the median or mean of TBL in each category. To reduce the effects of the relevant factors on the results, we preferred to extract and analyze the adjusted OR to unadjusted OR. If the study had several ORs which were adjusted for different combinations of relevant factors, we extract OR which was adjusted for the most number of relevant factors.

### 2.4 Quality assessment

Two authors (WW and BZ) independently assessed the included studies in our meta-analysis. The included studies were assessed by the Newcastle–Ottawa Scale (NOS).^[14]^ The NOS is judged on 3 broad subscales: the selection of the study groups contains 4 items; the comparability of the groups contains 2 items, and the ascertainment of the exposure or outcome of interest for observational studies contains 3 items. A maximum 9 scores could be given to the highest quality studies. A score of 5 or more was regarded as “high quality”; otherwise, the study was regarded as “low quality.”

### 2.5 Statistical analysis

This meta-analysis was conducted using the Stata software package (Version 12.0; Stata Corp., College Station, TX). We compared the difference of bilirubin between DN and NDN groups using the standardized mean difference (SMD) and pooled odds ratios (ORs). We used the chi-square-based Q-test to assess the heterogeneity among the individual studies. Heterogeneity was quantified based on *I*^2^, which ranged from 0% to 100% (*I*^2^ = 0% to 25%, no heterogeneity; *I*^2^ = 25% to 50%, moderate heterogeneity; *I*^2^ = 50% to 75%, large heterogeneity; *I*^2^ = 75% to 100%, extreme heterogeneity). If *I*^2^ was larger than 50%, a random effects model was used; otherwise, the fixed model was used.

If higher heterogeneity existed in our meta-analysis, subgroup analysis was used by study design, year of publication, gender, age, BMI, and duration of diabetes mellitus to find the source of heterogeneity. Sensitivity analysis was used to assess the robustness of the results in our meta-analysis. The purpose of sensitivity analysis was to evaluate the effect of a single study on the overall pooled estimates. In the sensitivity analysis, we excluded each study in turn and obtained the pooled estimates from the remaining studies. We assessed the possibility of publication bias using visual inspection of funnel plots and Egger's test. We also performed the Duval and Tweedie nonparametric “trim and fill” procedure to further assess the possible effect of publication bias in our meta-analysis. A 2-sided *P* value < 0.05 in the statistical process was considered statistical significant.

To evaluate the dose–response association between TBL and the risk of DN, we conducted the dose–response meta-analysis to calculate study-specific slopes (i.e., linear trends) and 95%CIs, which was proposed by Greenland and Longnecker and Orsini et al.^[15,16]^ If the study reported exposure category by a range, the midpoint was calculated by averaging the lower and upper bound; if the upper boundary for the highest category was not provided, the midpoint of the category was set at 1.5 times the lower boundary. When the lowest category was open-ended, we set the lower boundary to zero.

### 2.6 Ethical statement

As all the included studies were grounded on the previous publications, ethical statement was not necessary.

## 3 Results

### 3.1 Literature search and study characteristics

**Figure 1:**
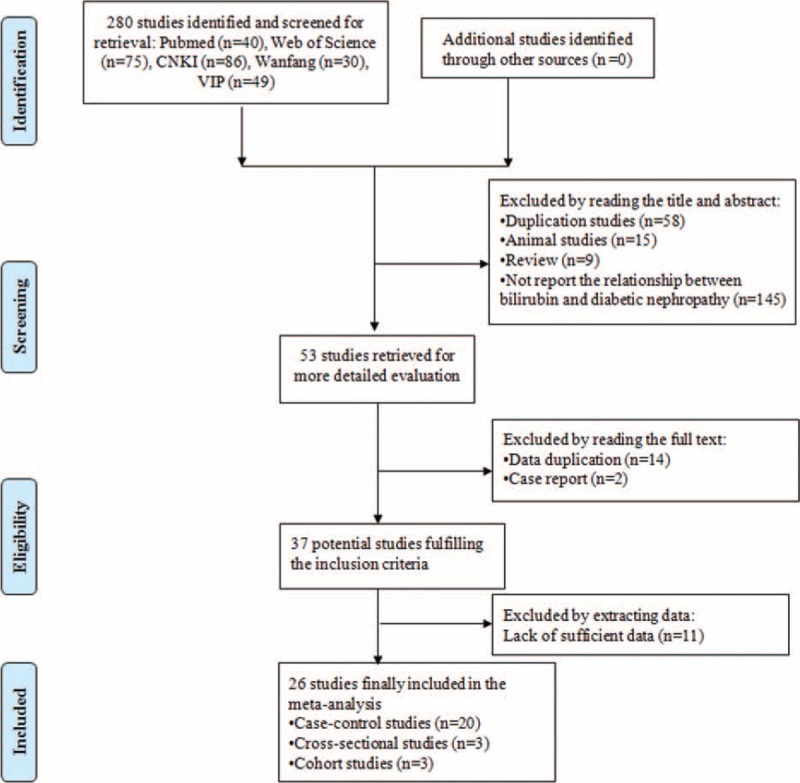
The process of study selection in our meta-analysis.

Figure 1 shows the study selection process in this meta-analysis. We identified 280 articles from 5 databases. In total, 227 articles were excluded after reading the title and abstract. However, 16 articles were excluded after reading the full text, which contained 14 duplication data articles and 2 case reports. After detailed evaluation, we excluded 11 articles, which did not report the mean value with standard errors or OR with 95% CIs or provide sufficient data to calculate them. Finally, 20 case-control studies,^[17–36]^ 3 cross-sectional studies,^[13,37,38]^ and 3 cohort studies^[12,39,40]^ were included in the meta-analysis, and 5 studies were included in the dose–response meta-analysis.

There were 23,141 subjects and 7944 DN patients in our meta-analysis. Twenty-three studies provided the mean values and their standard errors, and 11 studies provided ORs and their 95%CIs. Five studies provided the exposure category and ORs with their 95%CIs. The included studies were published between 2007 and 2016. The number of subjects ranged from 87 to 9795 and the duration of diabetes mellitus ranged from 5.27 to 25.41 years. The basic characteristics of the included studies in our meta-analysis were shown in Table 1 . The included 26 studies which were evaluated quality using the NOS were high quality.

**Table 1:**
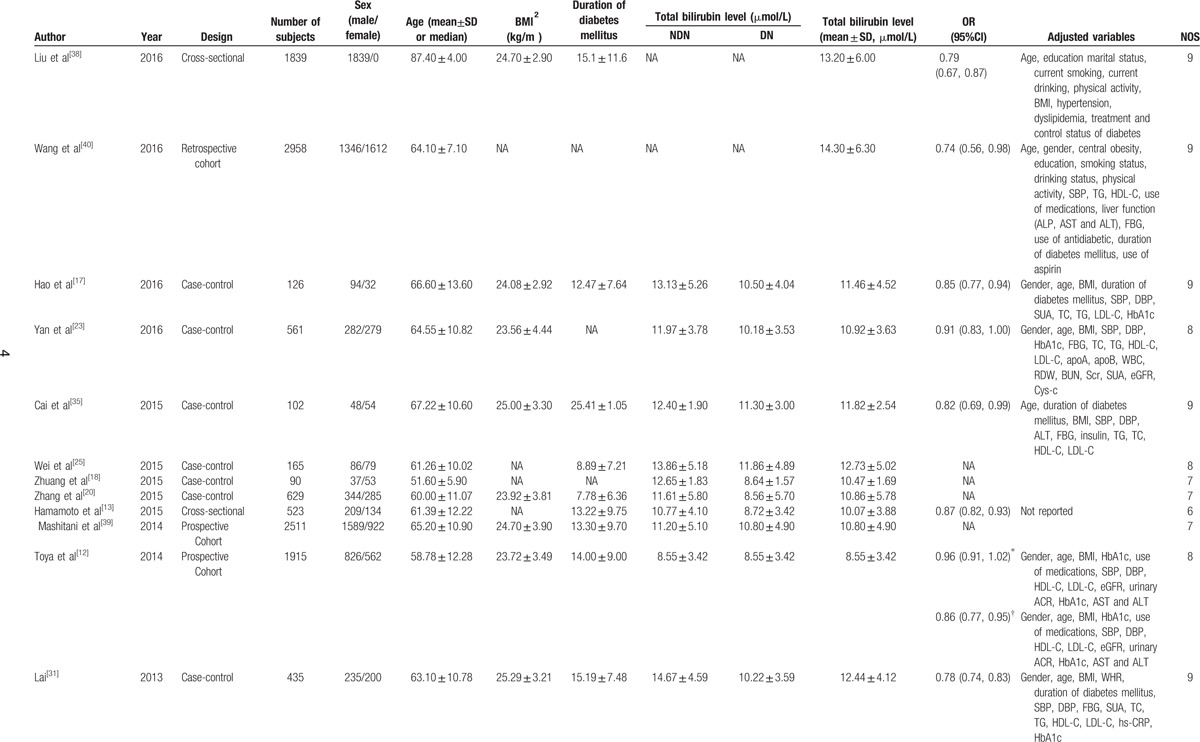
Characteristics of the studies included in the meta-analysis.

**Table 1 (Continued):**
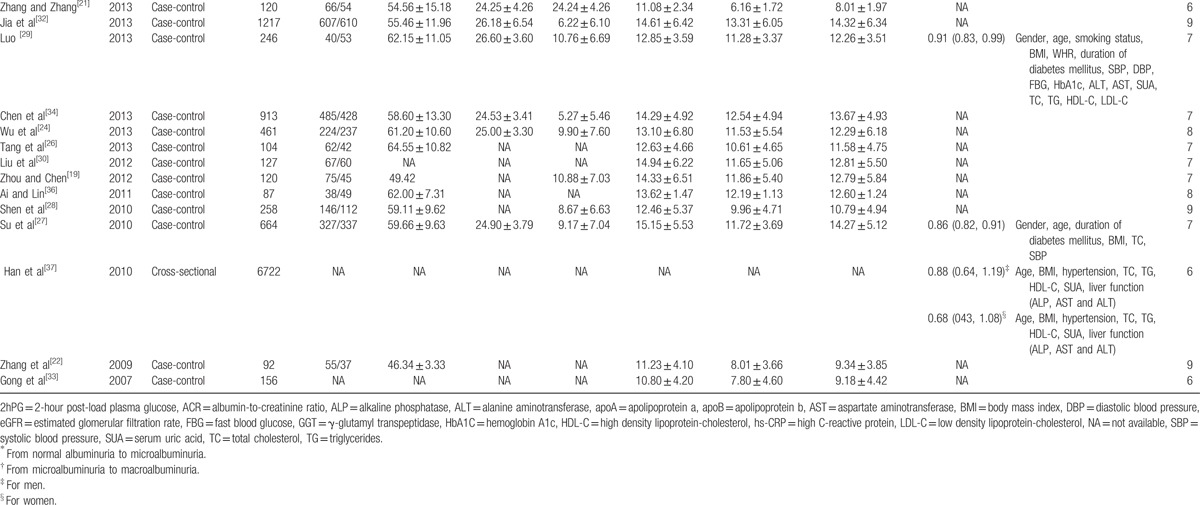
Characteristics of the studies included in the meta-analysis.

### 3.2 Overall and subgroup analysis

**Figure 2:**
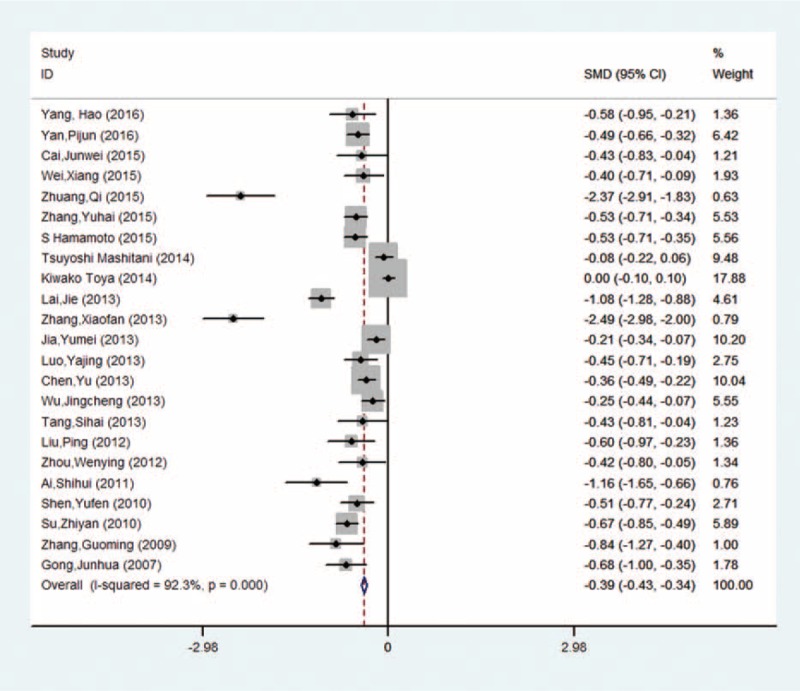
The SMD in the comparison between TBL in DN and NDN. DN = diabetic nephropathy, SMD = standard mean difference, TBL = total bilirubin level.

Figure 2 showed the results from the random-effects meta-analysis combining the pooled SMD. It was indicated that the TBL in the DN group was lower than that in the NDN group (SMD: −0.39, 95% CI: −0.43, −0.34), and there was an obvious heterogeneity among 23 studies (*I*^2^ = 92.3%); see Fig. 2. Therefore, we performed subgroup analysis and meta-regression to analyze the source of heterogeneity. Subgroup analysis was conducted by study design, year of publication, gender, age, BMI, and duration of diabetes mellitus; the result of each subgroup showed that TBL in the DN group was lower than that in the NDN group. The results of meta-regression indicated that the duration of diabetes mellitus might be the source of heterogeneity (Table 2).

**Table 2:**
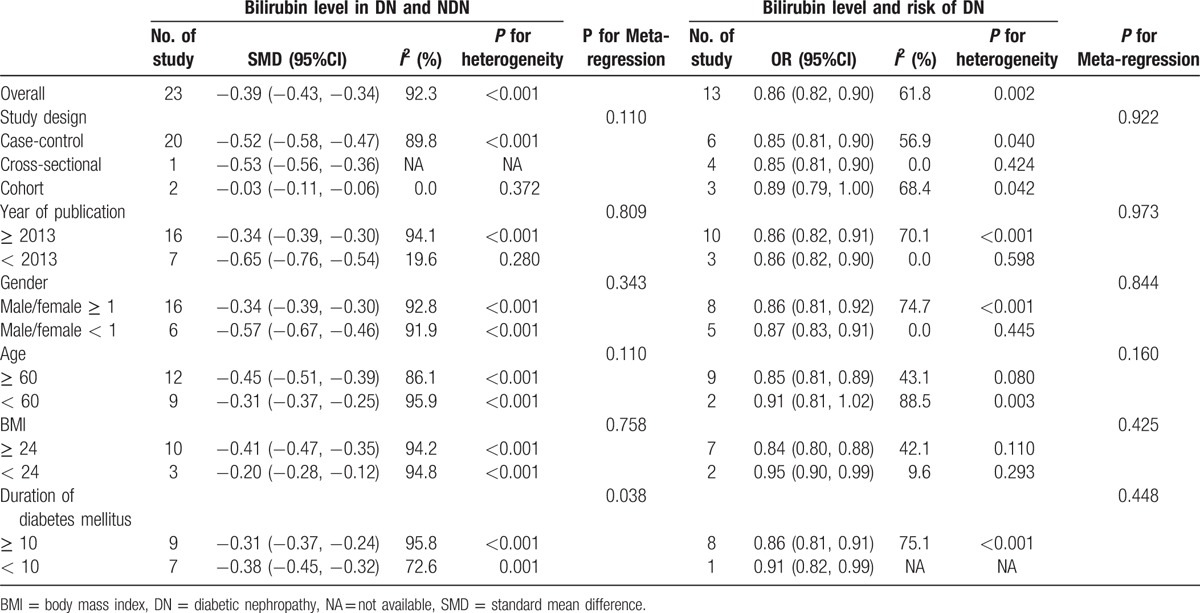
The standard mean difference (SMD) and pooled ORs on behalf of the relationship between the bilirubin level and the risk of DN.

**Figure 3:**
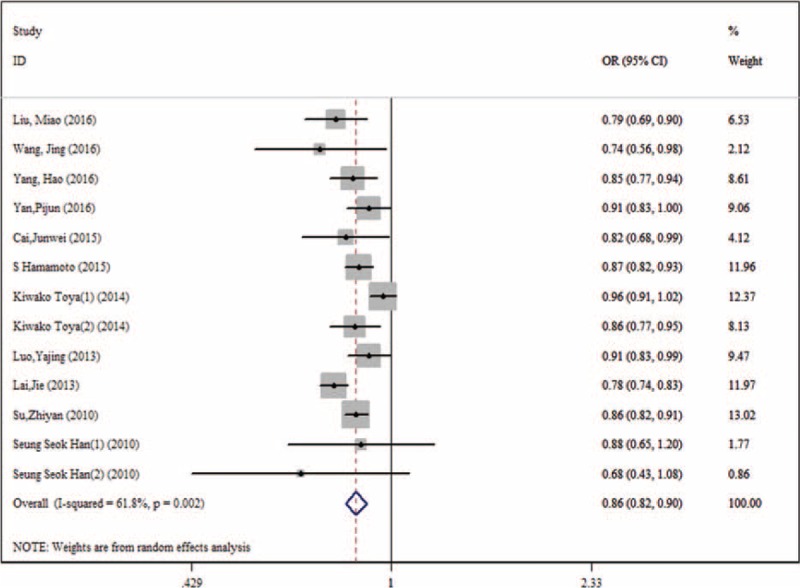
The pooled ORs in the effect of TBL on the risk of DN. DN = diabetic nephropathy, OR = odds ratio, TBL = total bilirubin level.

Figure 3 showed the results from the random-effects meta-analysis combining the ORs for DN in relation to TBL. It was indicated that there was a significant negative relationship between TBL and the risk of DN (OR: 0.86, 95% CI: 0.82, 0.90), and there was an obvious heterogeneity among 8 studies (*I*^2^ = 61.8%). The results of subgroup analysis were similar to those of SMD; no sources of heterogeneity were detected by meta-regression (Table 3).

**Table 3:**
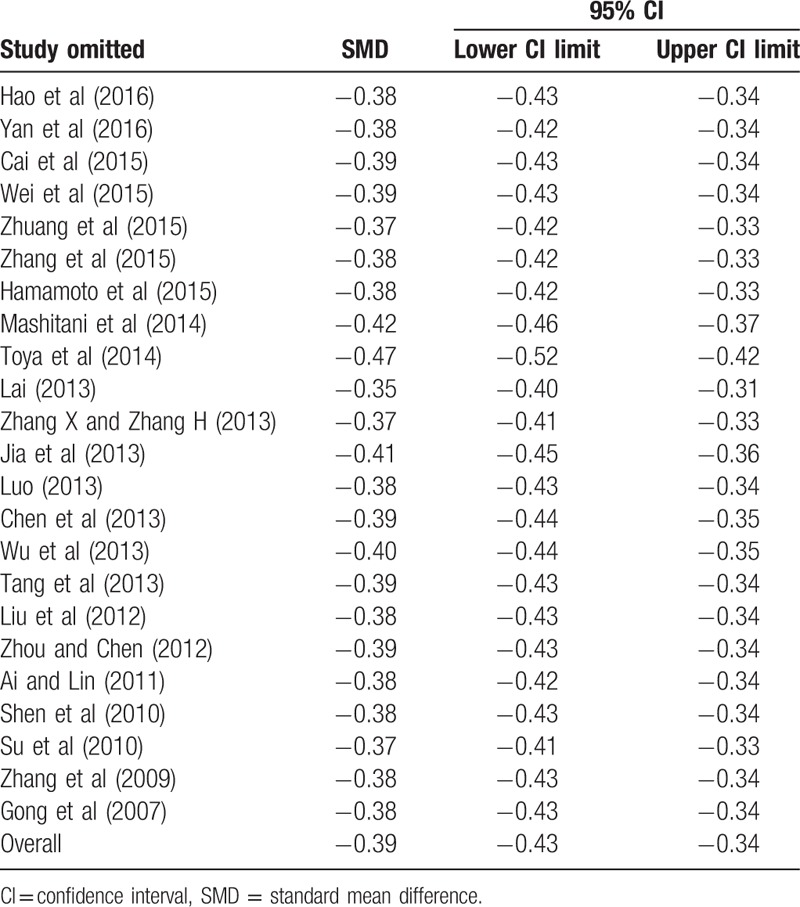
Sensitivity analysis on standard mean difference (SMD) by removing each study in each model.

### 3.3 Sensitivity analysis

Sensitivity analysis was carried out to assess the stability and reliability of the results. After dropping each single study from the pooled analysis, both the SMD and pooled ORs were not found to be affected (Tables 3 and 4).

**Table 4:**
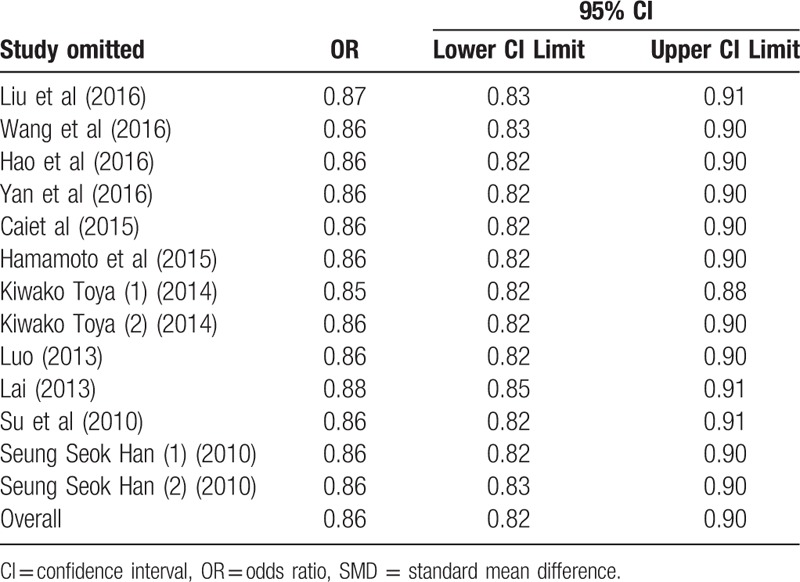
Sensitivity analysis on the pooled ORs by removing each study in each model.

### 3.4 Publication bias

**Figure 4:**
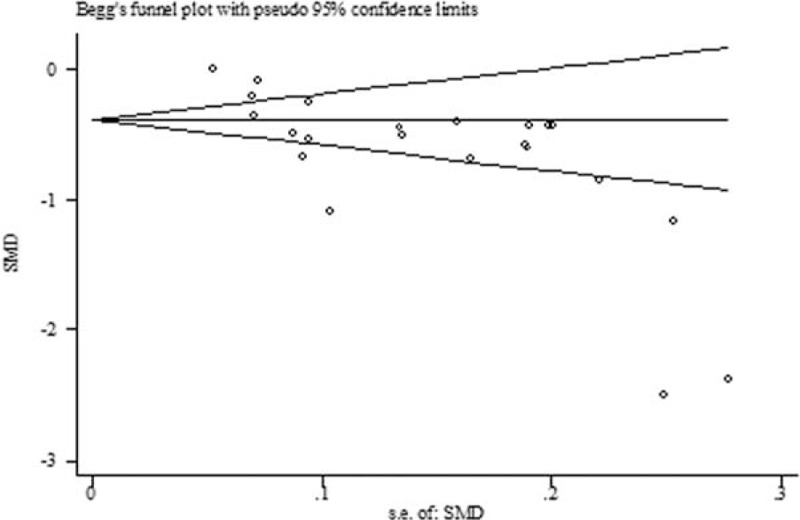
The trim and fill funnel plot in the comparison between TBL in DN and NDN. DN = diabetic nephropathy, TBL = total bilirubin level.

**Figure 5:**
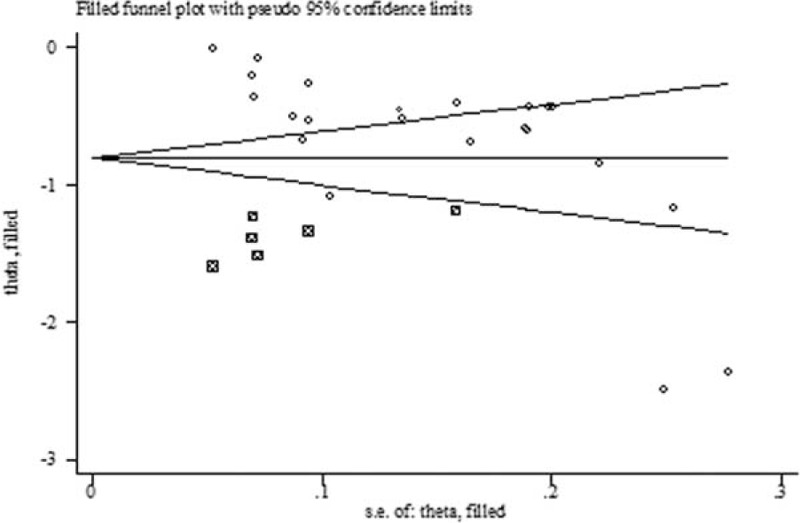
Begg's funnel plot in the effect of TBL on the risk of DN. DN = diabetic nephropathy, TBL = total bilirubin level.

Both Begg's funnel plot and Egger's test showed that publication bias existed in the comparison between TBL in DN and NDN (*P* for Begger's test = 0.008; *P* for Egger's test < 0.001) (Fig. 4). Thus, we perform the trim and fill method to identify and correct the asymmetry of the funnel plot; the result showed a significant association between TBL and the risk of DN (SMD: −0.81, 95% CI: −1.03, −0.58, *P* for heterogeneity < 0.001). Both Begg's funnel plot (Fig. 5) and Egger's test showed no publication bias in the effect of TBL on the risk of DN (*P* for Begger's test = 0.200; *P* for Egger's test = 0.367).

### 3.5 Dose–response relationship between TBL and the risk of DN

**Figure 6:**
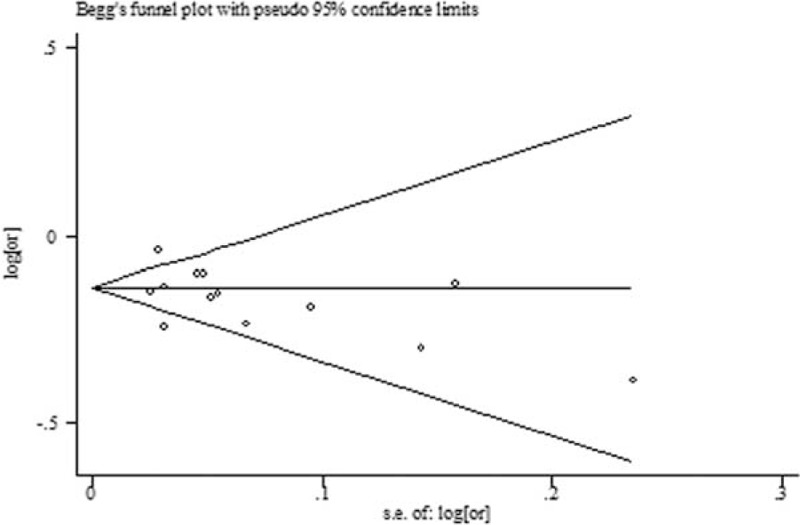
Begg's funnel plot in the comparison between TBL in DN and NDN. DN = diabetic nephropathy, TBL = total bilirubin level.

**Figure 7:**
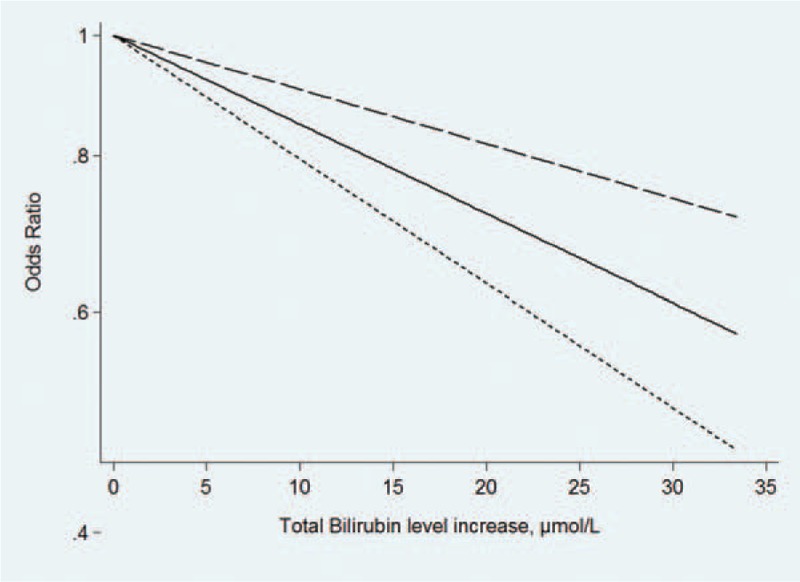
The dose–response analysis in the effect of TBL on the risk of DN. DN = diabetic nephropathy, TBL = total bilirubin level.

For dose–response analysis, we included 5 studies involving 14,391 subjects and 1693 DN cases which divided TBL into at least 3 exposure categories. We observed a linear association between TBL and the risk of DN (*P* = 0.156 > 0.05) (Fig. 6). A positive dose–response relationship exited between TBL and the risk of DN (the pooled OR was 0.75 [95%CI: 0.68, 0.84] per 17.1 μmol/L [1 mg/dL] increase in TBL), without significant heterogeneity across studies (*P* = 0.599, *I*^2^ = 15.9%) (Fig. 7).

## 4 Discussion

To our knowledge, this study was the first meta-analysis to assess the association between TBL and DN. In our meta-analysis, first, the results showed that TBL in the DN group was lower than that in the NDN group (SMD: −0.39, 95% CI: −0.43, −0.34), and the results were not affected by study design, year of publication, gender, age, BMI, and duration of diabetes mellitus. The results of meta-regression found that the duration of diabetes mellitus might be the source of heterogeneity. Second, our results also showed that there was a significant negative relationship between TBL and the risk of DN (OR: 0.86, 95% CI: 0.82, 0.90), the results of subgroup analysis were similar to those of SMD, and no sources of heterogeneity were detected by meta-regression. Sensitivity analysis did not find the stability and reliability of the results were not affected. We also found a positive dose–response relationship exited between TBL and the risk of DN and the pooled OR was 0.75 (95%CI: 0.68, 0.84) per 17.1 μmol/L (1 mg/dL) increase in TBL.

Bilirubin is the end product of heme catabolism by heme oxygenase (HO), especially HO-1. HO converts heme to biliverdin, and then biliverdin was reduced to bilirubin by biliverdin reductase. As a nonpolar molecule, bilirubin is solubilized in the vascular bed by binding to album, belongs to a phylogenetically old superfamily of tetrapyrrolic compounds, which have many biological functions, such as chronobiology, energy generation, transport, and homeostasis of oxygen by hemoglobins and myoglobin. Hemoglobin (Hb) degradation is the main source of bilirubin; it is possible that individual differences in bilirubin concentrations could be due to differences in the Hb concentration. We also found that an increase in red blood cell mass led to higher bilirubin levels.^[41]^ Thus, serum bilirubin may have a potential protective effect against oxidative damage, preventing the development and progression of oxidative stress-mediated vascular diseases.^[42]^ In recent years, several animal studies have indicated that bilirubin treatment protects against T2DM and its vascular complications.^[42–44]^ A number of observational studies had shown there was a negative relationship between TBLs and risk of T2DM and its vascular complications, but their results were inconsistent.^[44,45]^ In Japanese patients with T2DM, the risk of developing albuminuria was higher in the lowest quartile of serum TBL than that in the highest quartile of serum TBL (HR: 5.76, 95% CI: 1.65, 24.93).^[46]^ After adjusting for factors known to be associated with diabetic retinopathy, the prevalence was significantly lower among persons with the highest bilirubin quartile compared with those with the lowest quartile (OR: 0.25, 95%CI: 0.09, 0.72) or compared with those in the third lower quartiles (OR: 0.25, 95% CI: 0.11, 0.58).^[11]^

Possible mechanisms between bilirubin and diabetic nephropathy: hyperglycemia leads to mitochondrial superoxide overproduction in endothelial cells of both large and small vessels, the increased superoxide production causes the activation of 5 major pathways involved in the pathogenesis of complications.^[47,48]^ Five major pathways included polyol pathway flux, increased formation of AGEs (advanced glycation end products), increased expression of the receptor for AGEs and its activating ligands, activation of protein kinase C isoforms, and over activity of the hexosamine pathway.^[47]^ As an antioxidant, bilirubin can inhibit lipid peroxidation and attenuate LDL oxidation, and reduce the generation of reactive oxygen species.^[47]^ In addition, bilirubin also can prevent endothelial cell death in diabetic rats by deriving from HO enzyme activity or administering exogenously.^[49]^

To make our results reliable, we had made efforts in several aspects. First, in our meta-analysis, all the included 26 studies were better quality by using NOS. In order to prove the credibility of our results, we not only compared TBL between the DN group and the NDN group, but also analyzed the effect of bilirubin on DN and dose–response relationship between TBL and the risk of DN. All the results were found that there was a negative relationship between TBL and DN. Second, serum TBL is affected by many factors including sex, age, BMI, vascular disease, dyslipidemia, and so on^[44,50]^; we found that all the studies which contained OR were adjusted for potential confounders, which contained age, gender, BMI, and the relevant biochemical indexes, and we extracted OR which was adjusted for the most number of relevant factors. Third, we performed subgroup analysis and meta-regression to explore the source of heterogeneity. We found that duration of diabetes mellitus might be the source of heterogeneity, the incidence of DN increased with the duration of diabetes mellitus. The result of subgroup analysis was similar to the pooled SMD and OR, indicated that duration of diabetes mellitus had no effect on our pooled results. We also performed subgroup analysis by other factors (study design, year of publication, gender, age, BMI), and their results were still similar to the pooled SMD and OR. The sensitivity analysis and trim and fill method also indicated that the results were stable and reliable.

However, our meta-analysis also has some limitations. First, when we calculated the SMD, the publication bias was found. Therefore, we used the trim and fill method to solve the question and also found there was a negative association between TBL and DN. Second, all the included studies were carried out in Asia. This might lead to selection bias due to race. Chan et al^[51]^ studied on the relationship between TBL and the risk of diabetic amputation events in the Australia, and found that there was a significant difference in the association between bilirubin concentration and the risk of diabetic amputation events. The results suggested that in addition to Asian population, bilirubin might have an impact on the risk of DN in the population from other counties. Third, when we carried out the dose–response analysis, there were only 5 studies, including 1 prospective cohort study, 1 retrospective cohort study, and 3 cross-sectional studies. The number of studies was small in our meta-analysis especially in the cohort study, so large-sample, long-term prospective cohort studies were still needed to validate our results.

In summary, our study speculates that decreased TBL might increase the risk of DN. Therefore, bilirubin which is one of the indexes of hepatic function may be used as a biomarker of DN. It helps early diagnosis and effective therapeutic strategies on DN.
